# Aniline Derivatives Containing 1-Substituted 1,2,3-Triazole System as Potential Drug Candidates: Pharmacokinetic Profile Prediction, Lipophilicity Analysis Using Experimental and In Silico Studies

**DOI:** 10.3390/ph17111476

**Published:** 2024-11-02

**Authors:** Elwira Chrobak, Katarzyna Bober-Majnusz, Mirosław Wyszomirski, Andrzej Zięba

**Affiliations:** 1 Department of Organic Chemistry, Faculty of Pharmaceutical Sciences in Sosnowiec, Medical University of Silesia in Katowice, 4 Jagiellońska Str., 41-200 Sosnowiec, Poland; 2 Department of Analytical Chemistry, Faculty of Pharmaceutical Sciences in Sosnowiec, Medical University of Silesia in Katowice, 4 Jagiellońska Str., 41-200 Sosnowiec, Poland; bober@sum.edu.pl; 3 Faculty of Materials, Civil and Environmental Engineering, University of Bielsko-Biała, 2 Willowa Str., 43-309 Bielsko-Biała, Poland; mwyszomirski@ubb.edu.pl

**Keywords:** triazole, lipophilicity, RP-TLC, drug-likeness

## Abstract

**Background**: The triazole ring is an attractive structural unit in medicinal chemistry, and chemical compounds containing this type of system in their structure exhibit a wide spectrum of biological activity. They are used in the development of new pharmaceuticals. One of the basic parameters considered in the initial phase of designing potential drugs is lipophilicity, which affects the bioavailability and pharmacokinetics of drugs. **Methods**: The study aimed to assess the lipophilicity of fifteen new triazole derivatives of aniline using reversed phase thin layer chromatography (RP-TLC) and free web servers. Based on in silico methods, the drug similarity and pharmacokinetic profile (ADMET) of synthesized molecules were assessed. **Results**: A relationship was observed between the structure of the title compound, including the position of substitution in the aniline ring, and the experimental values of lipophilicity parameters (logP_TLC_). Most of the algorithms used to determine theoretical logP values showed less sensitivity to structural differences of the tested molecules. All obtained derivatives satisfy the drug similarity rules formulated by Lipinski, Ghose and Veber. Moreover, in silico analysis of the ADME profile showed favorable values of parameters related to absorption.

## 1. Introduction

Despite the discoveries in medicine and progress in pharmaceutical sciences that have been made in recent years, there is still a lack of effective methods for treating many diseases. Hence, there is a constant need to develop new therapies and drugs with high effectiveness and selectivity.

**Figure 1:**
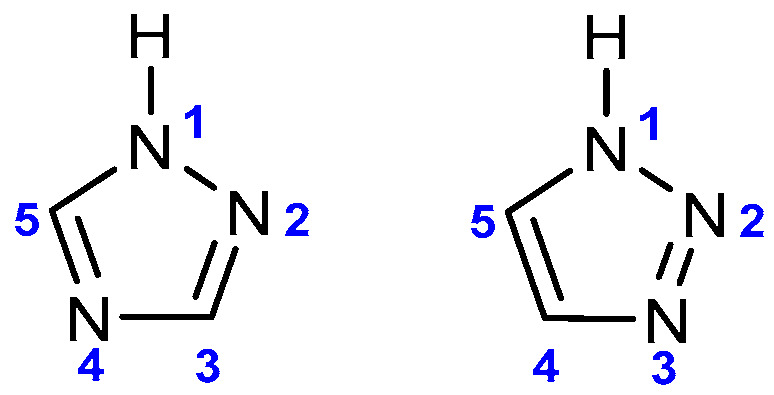
Scheme of triazole structures; 1,2,4-triazole and 1,2,3-triazole ring.

Often, the complex and time-consuming process of developing a new therapeutic agent is based on the analysis of substances and processes that are important for the functioning of the natural environment. An example of this is that of *N*-heterocyclic compounds, which are commonly present in nature and are an element of many physiologically important substances (e.g., nucleic acids and vitamins). In recent years, there has been growing interest in the synthesis of high nitrogen heterocyclic systems, including triazole derivatives, which are pharmacologically active substances with proven multi-targeted activity. These five-membered rings, depending on the position of the three nitrogen atoms, occur in the form of two isomers: 1,2,3- and 1,2,4-triazoles (Figure 1) [1].

**Figure 2:**
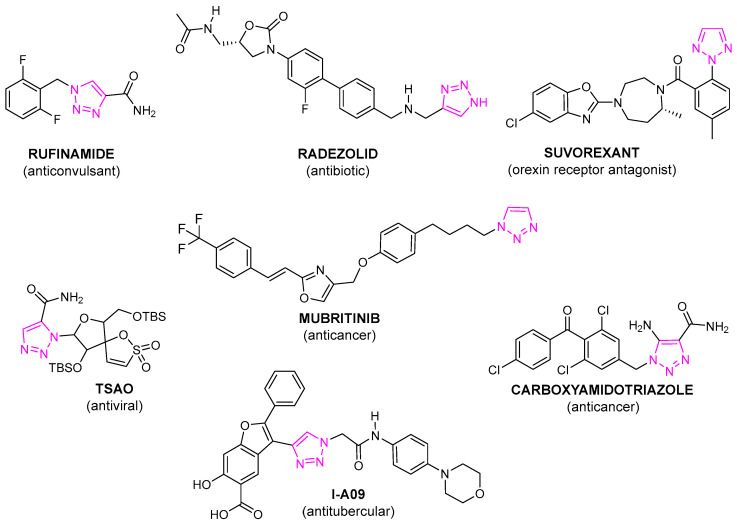
Selected compounds containing a 1,2,3-triazole ring used in therapy or clinical trials (1,2,3-triazole rings marked in magenta).

There are many drugs available in the market containing the 1,2,4-triazole ring, among which are fluconazole, voriconazole, efinaconazole, posaconazole (antifungal activity), letrozole, anastrozole (breast cancer treatment), ribavirin (antiviral activity), alprazolam (anxiolytic), rizatriptan (anti-migraine activity) and triazolam (hypnotic properties) [2,3]. The development of simple and efficient methods for the synthesis of 1,2,3-triazoles, allowing the control of the regioselectivity of the reactions forming their derivatives, has led to an increase in interest in this isomer [4]. Understanding the biological significance of 1,2,3-triazole derivatives has become a promising, dynamically developing field of research in medicinal chemistry. Compounds containing a 1,2,3-triazole fragment in their structure exhibit a wide spectrum of pharmacological activities [5], such as antifungal [6,7,8,9,10], anti-inflammatory [11,12], antiviral [13,14,15,16,17,18], antibacterial [19,20,21], antitubercular [22] and anticancer [23,24] activity. 1,2,3-Triazole systems are isosteric to the amide group, and can form hydrogen bonds and, due to their strong dipole moments, dipole–dipole interactions. These molecules are characterized by a high ability to affect a variety of biological targets, which determines the possibility of their use in the therapy of various diseases. Triazoles are also often used in medicinal chemistry as biological linkers in the synthesis of various types of hybrids and conjugates, which are investigated as potential therapeutics [25,26,27,28]. Despite many promising research results, only a few drugs containing 1,2,3-triazole ring have been approved for use in therapy so far (cefatrizine, tazobactam, rufinamide, suvorexant radezolid and mubritinib) [28] and several others are currently in clinical trials (carboxy-amido-triazole CAI, TSAO, I-A09) [29] (Figure 2).

It has been shown that the use of nitrogen-containing groups (pyridine, piperidine, morpholine, pyrrolidine, triethylamine, and dimethylethanolamine) to convert various organic compounds into ionic form can significantly improve their water solubility, thereby increasing their bioavailability and activity [30]. In recent years, there has been an increasing interest in the possibility of converting 1,2,3-triazoles into the corresponding triazolium salts with favorable chemical properties, such as high stability and diverse bioactivity [31].

As is widely known, the search for new medicinal products is a multi-stage and expensive process and is also burdened with a high risk of failure. Understanding the physicochemical properties of a drug candidate compound can increase the likelihood of effective delivery and therapeutic success [32]. The old approach to drug discovery was based on the assumption that greater potency in vitro would lead to more effective therapy. Nowadays, many companies focus on analyzing the properties of compounds at an early stage of preclinical development in order to mitigate more expensive clinical failures at a later stage [33,34].

The causes of this type of failure often include problems related to the pharmacokinetics of new active compounds or resulting from their toxicity. The development of in silico methods in recent years has proven to be a great support in the process of designing new drugs thanks to the possibility of predicting the pharmacokinetic profile, i.e., the properties of chemical substances related to the processes of absorption, distribution, metabolism, excretion and toxicity (ADMET) [35,36,37].

A property that plays a huge role in the process of drug absorption, distribution and transport in the body, and thus affects the pharmacodynamics of drugs, is lipophilicity. This is a physicochemical parameter that determines the behavior of a chemical compound in a two-phase system consisting of an organic phase (non-polar) and an aqueous phase (polar) [38].

Lipophilicity is determined theoretically. using various online tools, or experimentally. The most frequently used experimental methods for determining the lipophilicity of chemical compounds are reversed-phase (RP) chromatographic methods, i.e., high-performance liquid chromatography (RP-HPLC) and thin-layer chromatography (RP-TLC). The first one is characterized by high accuracy and low consumption of the tested compound, but the reversed-phase thin-layer chromatography (RP-TLC) method also has many advantages, including simple equipment and low analysis cost [39].

Determining the relationship between lipophilicity parameters and the biological properties of the analyzed compounds may help plan subsequent syntheses to obtain compounds with optimal biological properties.

Popular inference methods for data analysis based on lipophilicity and biological properties are Cluster Analysis (CA) and Principal Component Analysis (PCA). Cluster analysis is a method that allows the grouping of data, taking into account their similarities and differences [40]. CA is used in many fields of science and allows for a better understanding of the relationships between data. The principal component analysis led to a reduction in the number of original results, thus facilitating and simplifying computational analysis [41]. When the data describing a given system are characterized by different dimensions, most analysts use standardization to avoid the unfavorable impact of different units on the analyzed dataset.

In the search for new drug substances containing a 1,2,3-triazole ring, synthesis and studies of various hybrid compounds have been carried out. Studies of hybrid systems of triazoles combined with cyclic basic systems, which are pharmacophores or systems suitable for further transformations to obtain compounds with higher activity, have been described. These include benzaldehyde [42], 4-nitroimidazole/piperazinyl [43], flavones [44], quinazoline [45], chromene/cumarin, chalcone, indole, (epi)podophyllotoxin, quinoline/quinolone, pyridine, pyrimidine, steroid [46], azole [47] or chiral Schiff bases [48]. Systems combined with an aniline ring have not been described so far, nor has the dependence of their properties on the position of substitution in this ring been studied.

**Figure 3:**
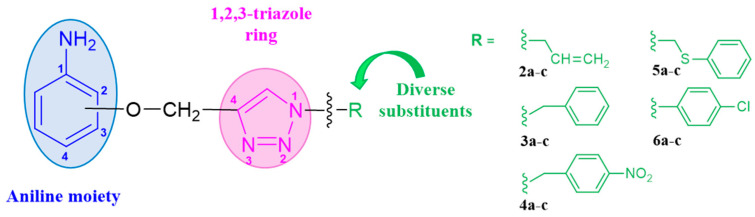
General structure of compounds synthesized for research (the position of substitution in the aniline ring is marked as follows: **a**—2 position, ortho-; **b**—3 position, meta-; **c**—4 position, para-).

The subjects of the research were 1,2,3-triazole derivatives connected at the C4 position through a methoxy linker with an aniline moiety (in different positions of the aromatic ring: 2, 3 or 4). The type of substituent located in the N1 position of the triazole ring also affects the properties of the target compounds [46]. In order to determine such a relationship for the structures studied in this work, a short carbon chain containing a double bond (allyl; **2a**–**2c**), diverse unsubstituted aromatic groups such as benzyl (**3a**–**3c**) and phenyl-thio-methyl (**5a**–**5c**), and substituted with an electron-withdrawing (nitro-benzyl; **4a**–**4c**) and electrondonating (chlorophenyl; **6a**–**6c**) group was selected (Figure 3).

The aim of this study was to compare the experimental and computational methods in the study of lipophilicity and to analyze the pharmacokinetic profile of fifteen triazole derivatives of aniline with potential biological activity using in silico methods.

## 2. Results and Discussion

### 2.1. Experimental Lipophilicity Determined by the RP-TLC Method

Lipophilicity is one of the most important properties determining the biological activity of medicinal substances because it affects the ability of the drug to be absorbed by biological membranes by passive diffusion [49]. Lipophilicity is expressed by the logarithm of the partition coefficient in the organic/aqueous phase system and has an impact on pharmacokinetics and pharmacodynamics, as well as toxicology. High lipophilicity (>5) often characterizes compounds with rapid metabolism, low solubility and poor absorption. Solubility is an important parameter from a pharmaceutical point of view, which depends on lipophilicity (log P) and, for small molecules, aqueous solubility is inversely proportional to lipophilicity [50]. Poor water solubility is also often the cause of poor absorption, and high concentrations of poorly soluble drug compounds in the body can cause their significant toxicity. Solubility is crucial for absorption and subsequent bioavailability of the drug in vivo. Bioavailability, most commonly studied as oral bioavailability, is a result of solubility, permeability and clearance, i.e., it directly or indirectly depends on lipophilicity. The optimal range of lipophilicity to achieve good bioavailability is a log P between 0 and 3, which is consistent with the range of lipophilicity in which a good balance between solubility and permeability can be achieved [51].

Assessing lipophilicity at an early stage of research on a potentially active molecule may help to design an optimal structure that increases the chances of success in clinical trials.

The experimental lipophilicity of derivatives **2**(**a**–**c**)–**6**(**a**–**c**) was examined by the RP-TLC method. This technique was used to determine the retardation factor R_f_ for each compound in five mobile phases consisting of acetone and Tris buffer in different volume ratios. The R_f_ values were converted into the chromatographic lipophilicity parameter R_M_ and then its normalized value R_M0_ was determined. Both of these parameters can be used to determine the lipophilicity of chemical molecules [52]. To characterize the tested compounds by logP, it was necessary to prepare a calibration curve. For this purpose, R_f_ values were determined for standard substances with a known logP_lit_ value using the RP-TLC method under conditions analogous to those for the tested substances. After calculating the R_M_ and R_M0_ values, the dependence of the logP_lit_ parameter on the experimentally determined R_M0_ values for the **A**–**G** standards was determined (Table 1).

This relationship is characterized by a high correlation coefficient (R = 0.989) (Figure S1, Supplementary Materials) and was used to calculate the logP_TLC_ parameter for the tested compounds **2**(**a**–**c**)–**6**(**a**–**c**) listed in Table S1 (Supplementary Materials).

logP_TLC_ = 1.0587R_M0_ − 0.0267

**Figure 4:**
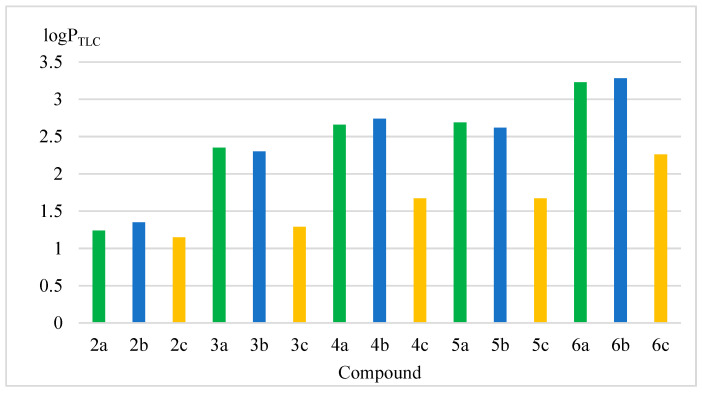
Graphical representation of experimentally determined logP_TLC_ parameter values for derivatives **2**(**a**–**c**)–**6**(**a**–**c**). The green color—indicates substitution in the ortho-position of the aniline ring, the blue color in the meta-position, the yellow color in the para-position.

The logP_TLC_ values of the tested compounds determined in this way, presented in Figure 4, range from 1.15 to 3.28. The exact values of the parameter for compounds **2**(**a**–**c**)–**6**(**a**–**c**) are given in Table S1 (Supplementary Materials).

Analyzing the obtained results depending on the position of attachment of the triazolo-methoxy substituent in the aniline ring, it can be seen that the lowest lipophilicity (logP_TLC_), in the range of 1.15–2.26, is demonstrated by para-substituted compounds: **2c**–**6c** (Table 2).

For ortho- and meta-substituted systems (**2a**–**6a** and **2b**–**6b**), the lipophilicity values are higher and comparable to each other.

In the case of compounds containing allyl, nitro-benzyl and chlorophenyl substituents, the highest lipophilicity values were characterized by those in which the aniline system was substituted in the meta position (**2b**, **4b**, **6b**). For the remaining substituents, the highest values were determined for aniline derivatives substituted in the ortho position (**3a**, **5a**).

Comparison of lipophilicity of the tested group of derivatives depending on the substituent introduced to the triazole ring (N1) showed that the lowest lipophilicity was exhibited by compounds **2a**–**2c** with an allyl substituent, which is the only one in its structure that does not contain an aromatic ring. The lowest logP value in this group was determined for the compound containing a substituted triazole system in the para position of the aniline system (**2c**).

The introduction of an additional aromatic substituent to the molecule clearly increases the lipophilicity of the obtained derivatives (compounds **3a**–**6c**). It is consistent with the literature data and is related to the change in the ratio of the number of aromatic atoms (sp^2^) to those with sp^3^ hybridization [53,54].

The increase in lipophilicity also depends on the method of connecting the aromatic system to the triazole ring. Among the tested compounds, we should also distinguish compounds **6a**–**6c**, in which the aromatic system (substituted in the para position with a chlorine atom) is connected by a direct bond to the N1 atom of the triazole ring. This method of connection stiffens the structure and increases lipophilicity. The highest logP values were determined for compounds **6a**–**6c**; from 2.26 (substitution in the para position) to 3.23 (in the meta position). Extending the linker connecting the triazole with the aromatic ring with a methylene group (-CH_2_-), as is the case in derivatives **3**(**a**–**c**)–**5**(**a**–**c**), reduces lipophilicity.

The introduction of a nitro group in the para position of the aromatic ring (compounds **4a**–**4c**) increases the lipophilicity compared to derivatives containing an unsubstituted benzyl system (**3a**–**3c**). The presence of a sulfur atom between the methylene group and the aromatic ring (without a substituent in the para position, compounds **5a**–**5c**) also increases lipophilicity compared to the benzyl derivative (**3a**–**3c**).

The lipophilicity of derivatives substituted in the para-position of the aniline ring differed significantly from ortho- and meta-substitution, which showed similar values. The weakest effect of the substitution position on the modification of the logP value was observed for allyl derivatives **2a**–**2c**. It can be assumed that the reason for the significant decrease in lipophilicity of para- substituted derivatives **3**(**a**–**c**)–**6**(**a**–**c**) is the greater rigidity of the system and the reduced possibility of intramolecular interactions between the amino group of the aniline and the additional aromatic ring introduced to the triazole moiety.

### 2.2. Theoretical Lipophilicity

As a result of the search for faster and more efficient methods of describing new, potentially biologically active molecules, online computational tools have been developed that allow for quick prediction of various parameters, including lipophilicity. This program works based on different algorithms based on the participation of atoms and larger fragments of particles or by considering their topology. The input data for the calculations are drawings of the structure of compounds or SMILES (*Simplified Molecular Input Line Entry Specification*) codes shown in Table S2 (Supplementary Materials), which constitute a clear record of the molecule structure. The values obtained in this way are presented in Table S3 (Supplementary Materials).

**Figure 5:**
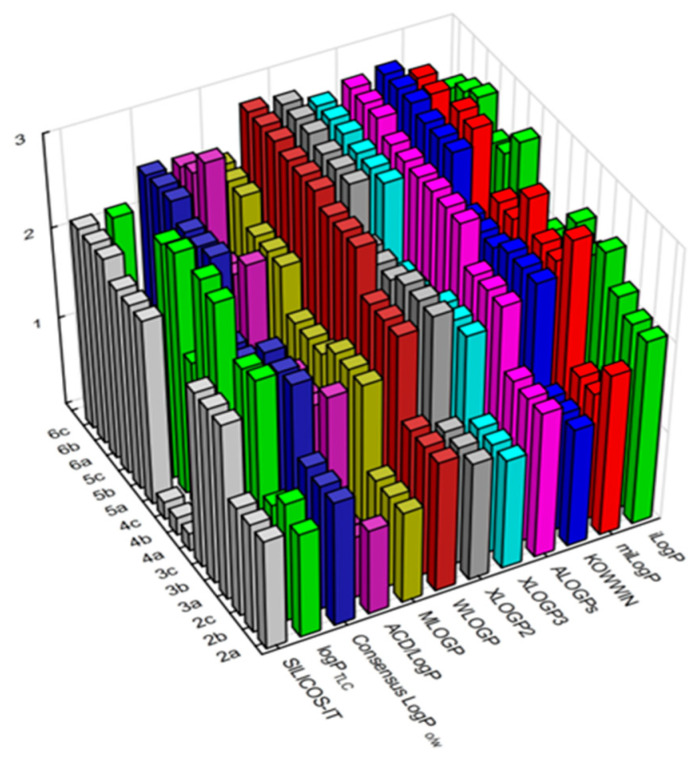
Graph of values of the lipophilicity parameters (logP_theor_ and logP_TLC_) determined for the tested compounds **2**(**a**–**c**)–**6**(**a**–**c**).

The experimental determination of the lipophilicity parameter was compared with theoretical values obtained using various online computational tools. They predict the logP values of new, not yet described structures of chemical compounds using computational algorithms based on the share of atoms or larger fragments and also by the use of the topology of molecules. To better present the differences in the results obtained, a graph of changes in lipophilicity determined by both techniques was prepared and presented in Figure 5. The experimentally determined lipophilicity parameter is marked with a light green color.

As can be seen, unlike experimental determinations (logP_TLC_), most theoretical methods do not take into account the influence of the substitution position of the aniline system. Only the iLOGP, miLOGP and ACD/LOGP programs predict such differences and, even though their nature is slightly different than the experimental results, they still show a general pattern of the highest lipophilicity value for ortho-substitution and the lowest for para-substitution. A significant deviation from the results obtained from the SILICOS-IT program is observed for derivatives containing a nitro group in the para position of the aromatic ring (compounds **4a**–**4c**).

### 2.3. Drug Likeness of Tested Compounds

Because a new chemical molecule is to be considered a drug candidate, it should meet certain criteria related to its pharmacokinetic, pharmacological and toxicological properties. In the process of developing a new drug, it is extremely important to initially assess the drug similarity of the new structure and examine its properties in terms of absorption, distribution, metabolism and excretion (ADME).

A preliminary assessment of “drug-like” properties of molecules is carried out early in the research process to accelerate the discovery and development of new drugs. There are various approaches to solving this problem. The simplest and most widely used was developed by Chris Lipinski and his colleagues at Pfizer and is generally referred to as Lipinski’s Rules or the Rule of Five [55].

The Rule of Five aims to determine whether a chemical compound with a specific pharmacological or biological activity has properties that make it an active drug when administered orally in humans. The criteria included in this rule are related to molecular properties relevant to the pharmacokinetics of a drug in the human body: absorption, distribution, metabolism and excretion (ADME). For a substance to meet Lipinski’s rule, it must have certain values (less than or equal to 5 or its multiples) of the following physicochemical properties: molar weight ≤ 500, number of nHA hydrogen bond acceptors ≤ 10, number of hydrogen bond donors nHD ≤ 5 and LogP ≤ 5 (or MlogP ≤ 4.15) [55].

The values of physicochemical parameters determined by the Rule of Five (Table 3) for the tested compounds **2**(**a**–**c**)–**6**(**a**–**c**) were compared with the ranges given by Lipinski.

The molar weight of the tested compounds **2**(**a**–**c**)–**6**(**a**–**c**) ranges from 230.27 (compounds **2a**–**c**) to 325.32 (compounds **4a**–**c**), so each of them meets the first criterion (M ≤ 500). In addition, the number of nHA hydrogen bond acceptors does not exceed 10. The tested compounds have 3 (**3a**–**c**; **6a**–**c**) or 5 (**2a**–**c**; **4a**–**c**; **5a**–**c**) acceptors. The third condition to be met is the number of hydrogen bond donors nHD lower than 5. Each of the tested compounds **2**(**a**–**c**)–**6**(**a**–**c**) has an nHD equal to 1. According to Lipinski’s rule, a drug-like compound should be characterized by appropriate lipophilicity (LogP ≤ 5 or MlogP ≤ 4.150) to ensure proper absorption. The experimentally determined values of the lipophilicity parameter logP (Table 3) for compounds **2**(**a**–**c**)-**6**(**a**–**c**) range from 1.15 to 3.28. The theoretically determined MlogP values presented in Table S3 (Supplementary Materials) for each tested compound **2**(**a**–**c**)-**6**(**a**–**c**) were less than 4.15 and ranged from 1.04 to 2.47. Therefore, we can say that the compounds we tested met all the guidelines formulated in the Rule of Five.

In addition to Lipinski’s rule, various other combinations of criteria have been described and used to predict the drug similarity of new biologically active chemicals. An example is Ghose’s rule, which changes the mass range of compounds to be between 160 and 480. Additionally, it sets the appropriate range of molar refraction values from 40 to 130 [56,57]. For the tested compounds **2**(**a**–**c**)–**6**(**a**–**c**), this rule was also met, and the molar refraction ranges from 65.81 to 89.98. Another rule was proposed by Veber, also taking into account the topological area of the polar surface of the molecule (TPSA) with values less than or equal to 140 Å^2^ [57,58]. Test compounds **4a**–**4c** and **5a**–**5c** were characterized by the highest TPSA values, equal to 111.78, while the lowest values were characteristic of compounds **2a**–**2c**, amounting to 65.81. Therefore, the compounds under study also met the physicochemical parameters of Ghose’s and Veber’s rules.

The effectiveness of a drug substance also depends on its solubility in water, which plays a key role in the method of drug administration. Hydrophobic drugs, poorly soluble in water, are difficult to dissolve in body fluids, which adversely affects their bioavailability and therapeutic effectiveness because it may lead to the binding of the drug in complexes with other lipophilic substances.

Using the SwissADME computing platform, LogS (ESOL), a parameter determining whether a substance is soluble in water, was determined. Compounds with LogS values in the range from −4 to −2 are defined as highly soluble; the lower the values are −4, the less they dissolve in water, and the higher the value −2, the higher their solubility in water [59]. For the tested compounds, the predicted LogS values ranging from −3.80 to −2.20, which allows them to be classified as water-soluble substances (Table 3).

### 2.4. In Silico Estimation of the ADME Profile of the Tested Compounds

Lipinski’s formulation of rules determining the similarity of a new chemical molecule to a drug initiated the intensive development of predictive methods allowing for a more detailed description of a potential drug at an early level of research. The most common reasons for rejecting a chemical molecule in the development process of a new drug are its unfavorable ADME profile and the associated poor bioavailability or the occurrence of undesirable side effects, i.e., both harm to the patient and toxicity to the environment [60]. The bioavailability of a drug determines its effectiveness and is related to its physicochemical properties, method of delivery, and the possibility of interacting with other substances and the processes of absorption, metabolism and excretion [61]. To predict the ADME profile of synthesized compounds, admetSAR, a free tool that is widely used in chemical and pharmaceutical research, was used [62,63] The results obtained in this way, determining the probability of various effects occurring, are presented in Table S4 (Supplementary Materials).

The first step that determines the fate of a drug in the human body is absorption, which depends on the drug used (form, chemical structure, degree of ionization or solubility) and the site of its administration. Most approved drugs are administered orally, and their absorption can occur in different parts of the gastrointestinal tract [61].

Human intestinal absorption (HIA) determines the drug’s ability to move through the intestinal epithelial barrier into the bloodstream, affecting its bioavailability [61]. ADME analysis determined that triazole derivatives **2**(**a**–**c**)–**6**(**a**–**c**) are compounds with good HIA. Assessment of absorption in the Caco-2 cell penetration model indicates that only compounds with a chlorophenyl substituent (**6a**–**6c**), regardless of the substitution site on the aniline ring, penetrate the intestinal barrier, while all other substituents located in the meta-position do not penetrate Caco-2.

The amount of drug that reaches the bloodstream after oral administration is considered as human oral bioavailability (HOB). Good HOB bioavailability is predicted for the synthesized compounds. The oral availability of a drug affects the dose required to achieve a pharmacological effect, which determines then the risk of side effects and toxicity of the drug [64].

Considering the distribution, the applied computational algorithms assessed the BBB (blood–brain barrier) penetration capacity for all tested triazole derivatives **2**(**a**–**c**)–**6**(**a**–**c**). This ability determines the potential usefulness of the drug for the treatment of diseases of the central nervous system (CNS), which may include neurological infections, Parkinson’s disease, Alzheimer’s disease and other neurodegenerative diseases. Such therapeutics, by penetrating the BBB, may interact with molecular targets in the CNS. In the case of other drugs, penetration through the BBB may cause adverse effects related to the CNS, e.g., neurotoxicity [65].

The proper functioning of the body requires continuous transport of various substances (xenobiotics and endogenous compounds) across biological membranes. Orally administered drugs dissolve, cross the intestinal wall, and travel with the blood to the liver, from where they enter the systemic circulation for distribution to tissues. On the other hand, drug elimination occurs through different routes: through the gastrointestinal tract (with bile), the kidneys (with urine) and the liver (as a result of metabolism). All these pharmacokinetic processes related to the crossing of different biological membranes are dependent on the physicochemical parameters of the drug but are also regulated by membrane transporters [66].

As a result of ADME analysis, they were shown to be both non-inhibitors and non-substrates for P-glycoprotein, a protein from the ABC transport protein family, transporting hydrophobic substances of neutral or cationic nature, but not transporting anions. PgP regulates the flow of xenobiotics (including drugs) between the cell and the environment, thus playing a protective role in cells against toxic compounds [67].

The group of organic anion-transporting polypeptides (OATPs) includes OATP1B1 and OATP1B3, located in the liver, and OATP2B1, present in the heart, kidney, placenta, lung, and intestine. The compounds tested are predicted not to be inhibitors of OATP2B1, but to inhibit OATP1B1 and OATP1B3. Inhibition of hepatocyte transporters can lead to clinically significant drug–drug interactions [68].

Organic cation transporters OCT2 and MATE1 (multidrug and toxin extrusion) are involved in the secretion of organic cations from the circulation into the kidney and then into the tubular lumen [69]. None of the tested compounds were classified as inhibitors of these transporters, while only the derivatives containing a sulphur atom (**5b**& selected as inhibitors of the bile salt export pump (BSEP)). BSEP is a transporter responsible for the movement of bile salts from hepatocytes to bile canaliculi, and its inhibition may lead to drug-induced liver damage due to the accumulation of these products in the liver [70].

Phase I metabolism of xenobiotics, including most drugs and toxins present in the environment, is catalysed by cytochrome P450 (CYP) enzymes. Drugs that are substrates are converted into metabolites by binding to the active site of the enzyme, while enzyme inhibitors can be its substrates or non-substrates. Common metabolic pathways dependent on CYP450 for two or more drugs cause drug–drug interactions and result from reversible or irreversible inhibition of CYP450. The largest share in drug metabolism is attributed to several of the many cytochrome isoforms (CYP3A, CYP1A2, CYP2C9, CYP2C19, CYP2D6). They are present primarily in the liver, but also in the intestinal wall [71].

According to literature data, triazoles are substrates and inhibitors of CYP. Cytochrome P450 enzymes are proteins containing heme and triazole-type rings that can interact with heme iron [72]. Analysis of parameters related to the metabolism of the tested compounds showed that only derivatives **3b**, **4a**–**c** and **6a**–**c** are substrates of the CYP3A4 isoform, while its inhibitors are compounds **3a**, **4a**, **5a**–**c** and **6a**–**c**. None of the compounds were classified as substrates of isoforms 2C9 and 2D6. For all compounds, the occurrence of property CYP450 inhibitory promiscuity is also predicted, which determines the ability of a drug or chemical substance to bind and reduce or weaken the activity of many different enzymes of the CYP450 isoforms.

The search for new compounds that are potential drugs aims to obtain molecules that, in addition to the expected biological activity, will be characterized by good bioavailability and high safety of use, i.e., the lowest probability of side effects and low harmfulness to the environment. A prediction of such properties was also made for the tested compounds. Table S5 (Supplementary Materials) summarizes selected parameters related to toxicity. According to the results, the tested compounds will not show carcinogenic effects (according to the binary model). Toxicity to kidney and liver cells was selected to evaluate the toxicity to human organs. All tested compounds may show hepatotoxicity, and only derivatives **2b**, **2c**, **5b** and **5c** were not classified as potentially nephrotoxic. No toxicity to terrestrial organisms, like honeybees, is expected; however, all compounds containing aromatic substituents attached to the **3**(**a**–**c**)–**6**(**a**–**c**) triazole system may be toxic to aquatic organisms.

### 2.5. Energies of the Highest Occupied Molecular Orbital (HOMO), Lowest Unoccupied Molecular Orbital (LUMO) and Dipole Moments as Calculated by DFTB+ Method

Nowadays, the analysis of frontier molecular orbitals (FMOs) has become a very significant method for the explanation of the electronic characteristics and reactivity of compounds. The transfer of electrons from the ground to the excited state mainly takes place from the FMOs, and one can also explain the kinetic stability and reactivity of the compounds from the energy difference of HOMO and LUMO (ΔE). The HOMO–LUMO gap relates to the kinetic stability of molecules. The larger the value of the gap, the higher the kinetic stability and the lower the chemical reactivity. This is related to the fact that adding an electron to the high-lying LUMO in order to remove electrons from a low HOMO is energetically unfavorable [73].

The Density Functional based Tight Binding (DFTB) method is based on a second-order expansion of the Kohn–Sham total energy in Density Functional Theory (DFT) with respect to charge density fluctuations. DFTB+ is the latest implementation of the DFTB method. It was used for calculation of HOMO and LUMO orbital energies and dipole moment values for all studied compounds. The results are presented in Table 4. The energy gaps between both frontier (HOMO and LUMO) orbitals are also presented there.

We observed that the HOMO orbital energy is always the lowest for meta- isomers and that the energy gaps for compounds of series **2**, **3** and **5** are very similar to each other except for compounds **4a**–**c** (which have the lowest value) and **6**. The meta- isomer energy gap is always the lowest for all compounds.

The highest occupied and lowest unoccupied molecular orbitals (HOMO and LUMO) in the molecule (especially the energy of the frontier orbitals, E_HOMO_ and E_LUMO_ or their difference ΔE) are considered to be important factors determining not only chemical reactivity but also biological activity [74,75].

**Figure 6:**
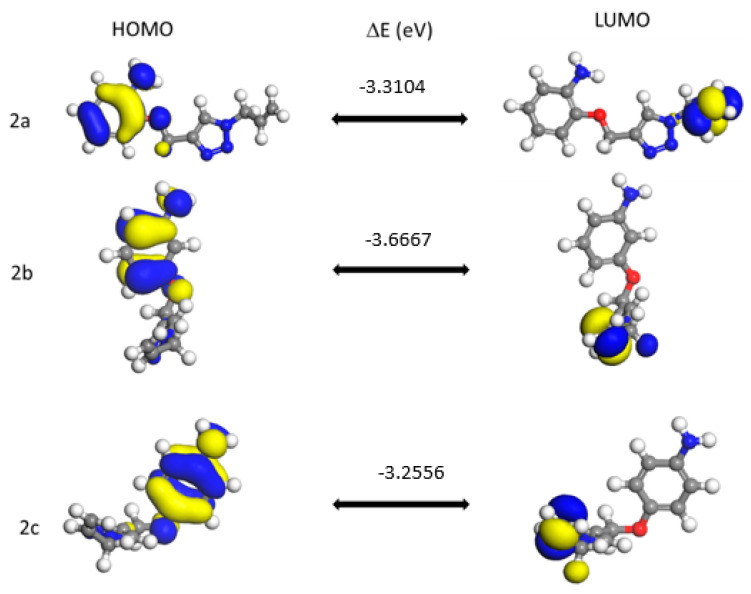
A graphic representation of both HOMO and LUMO orbitals of compound **2** *o*-, *m*- and *p*-isomers of with energy gap, ΔE (eV). The positive phase is denoted by blue, while the yellow color represents the negative phase of frontier orbitals.

Figure 6 represents the examples of the HOMO and LUMO orbitals of three *o*-, *m*- and *p*-isomers of compound **2**. The energy gap, ΔE (eV), between both frontier orbitals for each isomer is shown.

Similarly to compounds **2a**–**2c**, for all synthesized compounds, LUMO orbitals are mainly located in the substituent part of the triazole system. HOMO orbitals in the studied compounds are delocalized on the aniline ring (regardless of its substitution position) and the methoxy linker.

Analysing the literature data, it can be seen that many compounds show a correlation between biological activity and dipole moment, which can be used in the design of new, more potent compounds. In the studies of azole derivatives, in terms of antifungal activity, it was observed that compounds with larger dipole moments showed higher activity (lowest MIC—minimum inhibitory concentration values) [76].

For each set of three isomers (*o*-, *m*- and *p*-) of the studied compounds **2**(**a**–**c**)–**6**(**a**–**c**), the highest dipole moment was observed for ortho isomer (Table 4).

### 2.6. Chemometric Analysis

The correlation analysis was performed first for all lipophilicity data to evaluate the relationship between the experimentally determined value and the values obtained by theoretical methods. Table 5 presents the values of correlation coefficients for all the relationships studied.

The fewest statistically significant correlation coefficients are for the dependences with SILICOS-IT. The lowest value of the correlation coefficient was observed then (r = 0.209). The highest correlation coefficient characterized the dependence between XLOGP2 and MLOGP (r = 0.986). This value is because of the similarity of the algorithm used to calculate these theoretical lipophilicity values. Dependencies for which correlation coefficients are significant, i.e., for which *p* < 0.05, can be described by correlation equations. Those correlation equations that describe the dependence logP_TLC_ = f(logP_theor._) (last column) are consequential, especially for our work. These equations could be of use to calculate the lipophilicity values of many compounds without the need to conduct an experiment in the laboratory. The highest correlation coefficient obtained for these dependencies describes the dependence between logP_TLC_ and the ACD/LogP value, which is 0.789. The obtained equations and the parameters describing them, except for the dependencies from iLOPG and SILICOS-IT, are presented in Table 6 due to low and statistically insignificant correlation coefficients.

**Figure 7:**
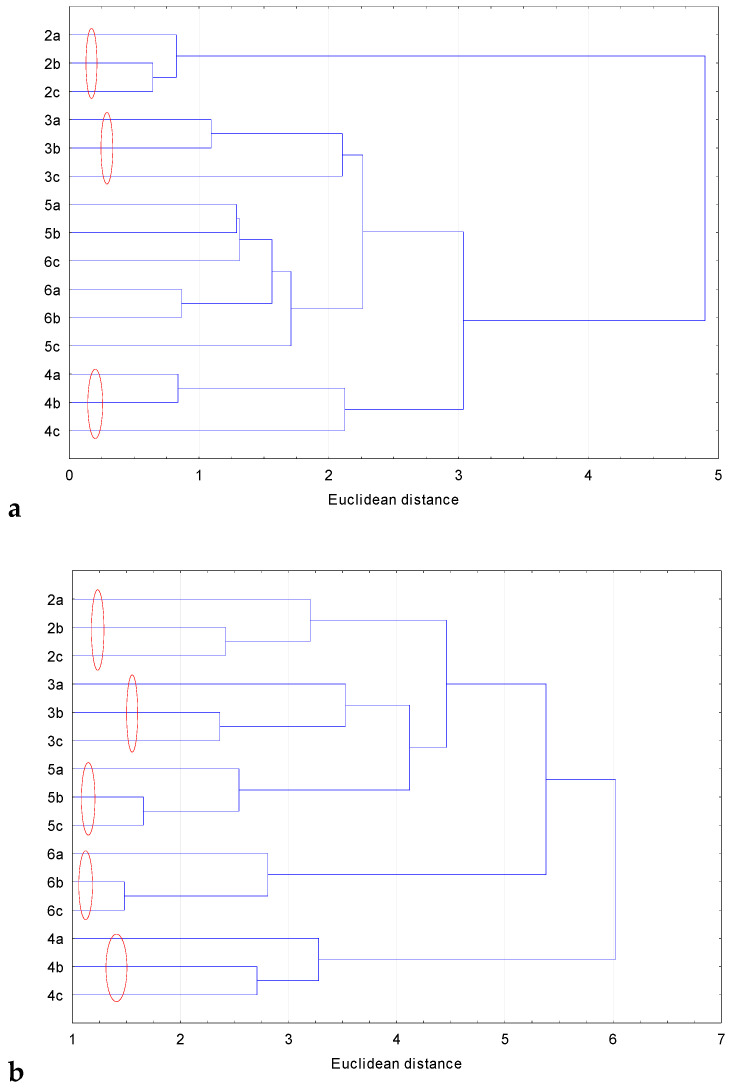

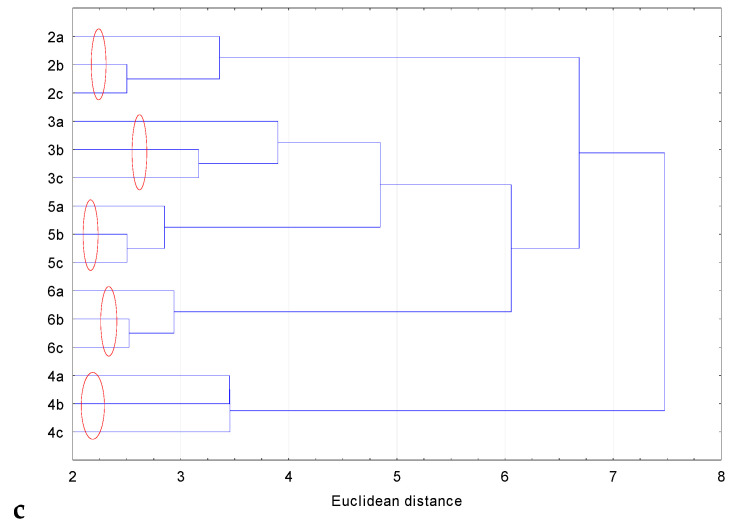
Cluster analysis based on lipophilicity values describing compound similarities (**a**), based on ADME values describing compound similarities (**b**) and compound similarities based on all values obtained (**c**). Red circles describe the clusters obtained.

Cluster analysis (CA) performed next included all analyzed values for all tested compounds. The analysis was carried out several times to find similarities between the compounds based separately on the values of their lipophilicity and pharmacokinetic properties and the basis of all data. All dendrograms are shown in Figure 7a–c.

As can be seen from Figure 7, the clusters obtained on the basis of the lipophilicity value (Figure 7a) and on the basis of the ADME value (Figure 7b) do not differ much but. in the second case, the grouping looks slightly different. In both cases, the clusters are formed by compounds **2a**, **2b** and **2c**; **3a**, **3b** and **3c** and **4a**, **4b** and **4c**. Thus, both in the case of lipophilicity and ADME values, it turns out that, regardless of the place of the substituent in the basic structure, the analyzed values are similar for individual groups of compounds and, in some cases, even the same. However, the difference in the analyses presented in Figure 7b,c is that the ADME values also group other derivatives, i.e., **5a**, **5b** and **5c** and **6a**, **6b** and **6c**. This proves that in the case of derivatives **5** and **6**, the place of attachment of the substituent does not influence the ADME data value. The lipophilicity values cause compounds **5c** and **6c** to exchange places in the obtained clusters. Table S3 (Supplementary Materials) clearly shows that most lipophilicities calculated using in silico methods have the same values regardless of the place of the substituent. This is because of the algorithm used for calculations for each calculation method. The exceptions are iLOGP, miLogP and ACD/LogP. Figure 7c shows the cluster analysis for all data obtained for the tested compounds, i.e., experimental and theoretical lipophilicity and pharmacokinetic parameter values. Figure 7c looks almost identical to Figure 7b, so the clusters group compounds according to substituents. This can be because pharmacokinetic properties influence cluster formation more than lipophilicity.

**Figure 8:**
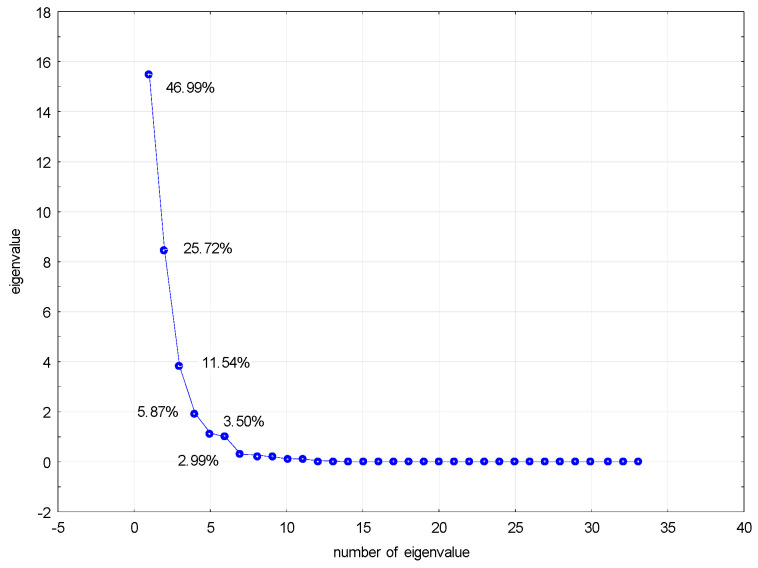
Scree plot for all compounds based on the data obtained.

The PCA was performed, too, for all analyzed data obtained for compounds **2**–**6**. The analysis was possible because the data describing the system, i.e., the data characterizing the analyzed compounds, show some correlations. The analysis allowed, based on the scree plot, the selection of six principal components and the formulation of further conclusions. The selected six principal components let us describe 96.6% of the variability of the system. The remaining eigenvalues are definitely less than unity. The figure below shows the scree plot obtained (Figure 8).

**Figure 9:**
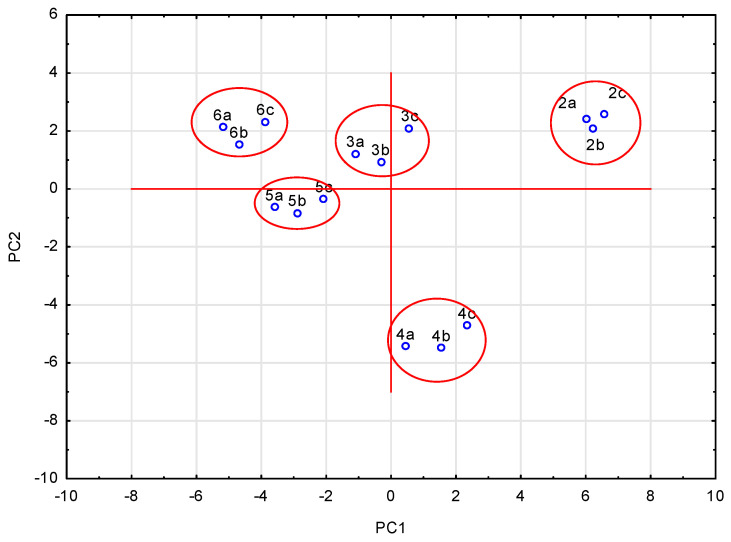
Projection of cases onto the factor plane for six principal components.

As we can see, the PCA analysis allowed for a significant reduction in the amount of data describing the examined system of variables. Individual principal component group variables are similar to each other. In turn, principal components do not correlate with each other. Data describing a given system are selected, then, but often in much smaller numbers than the original dataset. By reducing the amount of data, it is also possible to classify the compounds studied. Based on these six obtained principal components, a graph of the projection of cases onto the factor plane can be drawn. This graph is shown in Figure 9.

It can be seen that the tested compounds were classified depending on the substituent used to modify the basic structure of the compound. The result of this analysis is the same as that of the previously performed cluster analysis. While the cluster analysis based only on the lipophilicity values for the tested compounds gave only three clusters combining compounds of the same group (compounds **2**, **3** and **4**), the cluster analysis based on the ADME values, and also on all values describing the compounds, gave clusters as shown in Figure 9. The conclusion is that reducing the amount of data does not change the analysis results.

## 3. Materials and Methods

### 3.1. Triazole Derivatives

**Figure 10:**
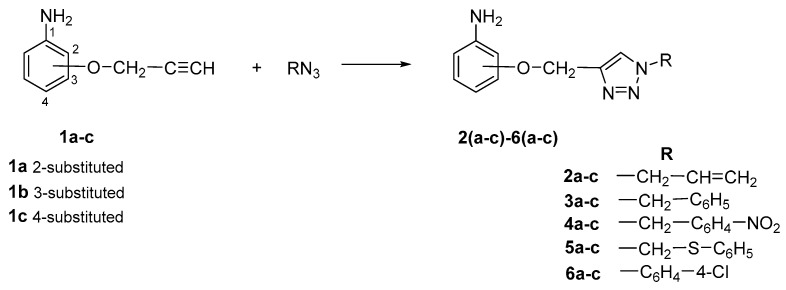
Scheme of synthesis of the tested triazole derivatives **2**(**a**–**c**)–**6**(**a**–**c**).

The present study used a series of triazole derivatives of aniline synthesized according to previously described procedures. The appropriate aniline propergyloxy derivatives **1a**–**1c** were obtained by alkylation of isomeric *o*-, *m*- and *p*-hydroxyacetanilides with propargyl bromide in DMF solution in the presence of sodium hydride and then deblocking the amino group by heating in a solution of hydrochloric acid in ethanol [77]. The synthesis of target triazole derivatives was carried out in a mixture of dimethylformamide and water in the presence of a copper catalyst (CuSO_4_ × 5H_2_O, sodium ascorbate) using azides with different structures (Figure 10) [78]. All reagents used in the synthesis were purchased from Merck (Darmstadt, Germany).

The derivatives **2**(**a–c**)–**5**(**a–c**) and **6b**,**c** were obtained in good yields ranging from 52 to 75%. To confirm the structure of the synthesized derivatives, ^1^H and ^13^C-NMR spectroscopy and HR–MS spectrometry techniques were used. NMR spectra were recorded using a Bruker Avance III 600 spectrometer (Bruker Corporation, Billerica, MA, USA) and HR-MS spectra using a Bruker Impact II instrument (Bruker Corporation, Billerica, MA, USA).The obtained results were consistent with those previously published [78].

Derivative **6a** was synthesized according to procedure C described by Kisiel-Nawrot et al. [78] and purified by column chromatography. Silica gel 60 (0.063–0.200 mm, Merck, Darmstadt, Germany) was used as the stationary phase, the eluent was chloroform (Merck, Darmstadt, Germany) and chloroform/ethanol (Merck, Darmstadt, Germany) showed a volume ratio of 10:1.

1-(4-chlorophenyl)-4-(2-aminophenoxy)methyl-1H-1,2,3-triazole **6a**:

Yield: 69%; ^1^H NMR (CDCl_3_, 600 MHz), δ (ppm): 3.84 (s, 2H, NH_2_), 5.33 (s, 2H, CH_2_), 6.75–6.77 (m, 2H, H_arom_), 6.86 (t, 1H, H_arom_), 6.96–6.98 (d, ^2^*J* = 7.8 Hz, 1H, H_arom_), 7.50–7.52 (d, ^2^*J* = 9 Hz, 2H, H_arom_), 7.69–7.70 (d, ^2^*J* = 9 Hz, 1H, H_arom_), 8.04 (s, 1H, CH); ^13^C NMR (CDCl_3_), 150.9 MHz), δ (ppm): 62.37 (CH_2_), 112.48, 115.53, 118.56, 120.85 (=C-H), 121.74, 122.12, 129.99, 134.69, 135.42, 136.61, 145.30 (C=C-H), 145.80; ESI-HRMS Calcd for C_15_H_14_ClN_4_O ([M + H]^+^): 301.0856, found: 301.0852.

### 3.2. Experimental Lipophilicity Determined by Reversed-Phase Thin-Layer Chromatography (RP-TLC)

In the process of determining the lipophilicity of the synthesized compounds, two groups of reagents were used. The first were reference substances whose lipophilicity (logP) had been previously determined and described in the literature; the second were solvents. Compounds with logP values in the range of 0.64 to 4.79 were selected as standards: benzamide (0.64), acetanilide (1.21), prednisone (1.62), 4-bromoacetophenone (2.43), benzophenone (3.18), anthracene (4.45) and dibenzyl (4.79) [79,80]. These substances with a purity of 98–99% were purchased from Merck (Darmstadt, Germany). Ethanol (99%, Merck, Darmstadt, Germany) was used as a solvent to obtain solutions (2 mg/1 mL) of standards and test substances. Eluents were prepared from acetone (99%, Merck, Darmstadt, Germany) and (tris-hydroxymethyl)aminomethane (0.2 M, pH = 7.4) Merck (Darmstadt, Germany) in a volume of 60 mL in the proportions 80:20, 70:30, 60:40, 50:50 and 40:60; *v*/*v*. The determinations were performed three times for each tested compound, in all development systems on chromatographic plates (7 cm × 10 cm), covered with modified silica gel RP-18F_254_S (Merck, Darmstadt, Germany). Chromatograms were visualized under UV light at wavelengths of 366 and 254 nm. For each compound, the distance traveled by the compound (D_comp.)_ and the distance of the mobile phase (D_m.phase_) front were measured in cm and the retardation factor R_f_ were calculated according to the following formula:

$$R_{f}=\frac{\mathrm{Dcomp}.}{D\;m.\mathrm{phase}}$$

Based on the obtained values, calculations were made of the chromatographic lipophilicity coefficient R_M_ defined by Bate-Smith and Westall [81].

$$R_{M}=\log\left(\frac{1}{R_{f}\;}-1\right)$$

The R_M_ values determined for each tested compounds **2**(**a**–**c**)–**6**(**a**–**c**) in various development systems were used to determine the linear dependence on the concentration of the organic component in the mobile phase. By extrapolating the composition of the mobile phase to a system containing only an aqueous component (concentration of acetone as an organic component equal to 0), the relative lipophilicity parameter R_M0_ was determined for the tested compounds.

### 3.3. Computational Programs Used to Determine Theoretical Lipophilicity and Selected Physicochemical and Pharmacokinetic Parameters

The online tool SwissADME (for iLOGP, XLOGP3, WLOGP, MLOGP, SILICOS-IT and Consensus LogP_o/w_) was used to predict most theoretical lipophilicity parameters, obtaining results based on SMILES codes [57]. Using the EPIWEB 4.1 program, the KOWWIN parameter was also obtained based on the SMILE code. miLogP values were calculated based on the structural formula [82], ALOGPs, XLOGP2 [83] and ACD/LogP [84]. The drug-likeness parameters of the compounds **2**(**a**–**c**)–**6**(**a**–**c**) were calculated using SwissADME (Swiss Institute of Bioinformatics, Lausanne, Switzerland) [57,59]. The absorption, distribution, metabolism, excretion and toxicity (ADMET) properties of synthesized compounds were examined using admetSAR 2.0 [85].

DFTB+ (version 22.1) [86], a software package for efficient approximate density functional theory based atomistic simulations was used for quantum calculations. After optimizations of geometry, energies of the highest occupied molecular orbital (HOMO), lowest unoccupied molecular orbital (LUMO) and dipole moment values were obtained.

### 3.4. Cluster Analysis and Principal Component Analysis

Both analyses—CA and PCA—were performed using STATISTICA 13 software. In the case of cluster analysis, all data regarding the analyzed compounds were considered, based on Euclidean distance [87]. The results were presented in the form of dendrograms. In the case of PCA, the value of OATP 2B1 was omitted because it has the same value for all compounds. The number of principal components was selected after analysis of the scree plot. For both CA and PCA, the data were standardized.

## 4. Conclusions

The titled 1,2,3-triazole derivatives synthesized on the basis of aniline molecules were analyzed based on their lipophilicity and ADME parameters. Lipophilicity values were determined not only theoretically but also experimentally. Analysis of experimentally obtained lipophilicity values showed that, considering the place of attachment of the triazole ring to the aniline system, the lowest logP_TLC_ values were found for compounds substituted in the para-position, regardless of the type of substituent in the N1 position (compounds **2c**–**6c**). Although the input data for all calculation programs are SMILES codes (notation unambiguously representing the structure of chemical compounds using ASCII character sequences), different for individual isomers (*o*-, *m*-, *p*-), the values of the lipophilicity parameter calculated by most programs were much less diverse than those obtained experimentally. Analyzing the lipophilicity determined using available algorithms, it was found that only the iLOGP, milogP and ACD/LOGP values differentiate the position of the aniline ring substitution.

All analyzed compounds meet the Lipinski rule, which is influential for potentially new drugs. Among other significant parameters, except for Lipinski’s rule, the analyzed compounds follow Ghose’s and Veber’s rules. The solubility of the compounds in water was also taken into account, and it turned out that all of them were very soluble, which is also an advantage for the drug, and indicates better bioavailability and therapeutic effectiveness. Imperative parameters in the design of new medications are those determining absorption, distribution, metabolism, excretion and toxicity. In the case of the analyzed compounds, all of them are characterized by good human intestinal absorption and human oral availability.

The conducted chemometric analysis for all analyzed properties (lipophilicity and ADME) indicates the similarity of the obtained compounds in terms of the substituent introduced into the main structure. The analysis is also irrelevant to the amount of data analyzed because the principal components obtained as a result of PCA analysis group compounds in the same way as cluster analysis (CA).

Selected compounds (**2a**, **3c**, **4b**, **4c**) were tested for anticancer activity but were not active against the cell lines used. The IC_50_ values determined for these derivatives against human glioma (SNB-19), lung cancer (A549) and breast cancer (T47D) cell lines were higher than 100µM [66]. Taking into account the promising characteristics of the tested compounds as potential drugs (including appropriate lipophilicity and water solubility), and, on the other hand, their lack of anticancer activity, further work should be focused on examining other directions of action. The presence of the triazole system in numerous biologically active molecules determines, among others, antibacterial and antifungal properties. For the compounds described in the work, tests are planned on reference gram-positive and gram-negative strains and representatives of multidrug-resistant clinical isolates (methicillin-resistant *S. aureus* (MRSA) and vancomycin-resistant *E. faecalis*).

## Figures and Tables

**Table 1:** Lipophilicity parameters for standard substances **A**–**G**.

Lipophilicity Parameters	Standard Substances						
	A Benzamide	B Acetamide	C Prednisone	D 4-Bromo-acetophenone	E Benzophenone	F Anthracene	G Dibenzyl
logP_lit_	0.64	1.21	1.62	2.43	3.18	4.45	4.79
R_M0_	0.9499	1.2020	1.6729	2.4678	2.7252	3.9213	4.8432
−*b*	0.0167	0.0228	0.0302	0.0340	0.0365	0.0488	0.0608
*r*	0.9902	0.9988	0.9933	0.9947	0.9979	0.9947	0.9910
logP_TLC_	0.66	1.25	1.74	2.58	2.86	4.12	5.10

**Table 2:** Experimentally determined lipophilicity logP_TLC_ (division of compounds taking into account the type of substituent and the places of its attachment).

Structure of Compound	Ortho- (2)		Meta- (3)		Para- (4)	
	No.	logP_TLC_	No.	logP_TLC_	No.	logP_TLC_
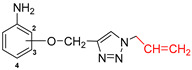	**2a**	1.24	**2b**	1.35	**2c**	1.15
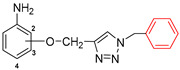	**3a**	2.35	**3b**	2.30	**3c**	1.29
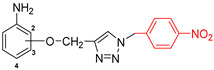	**4a**	2.66	**4b**	2.74	**4c**	1.67
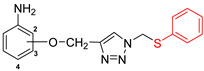	**5a**	2.69	**5b**	2.62	**5c**	1.67
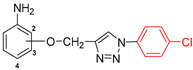	**6a**	3.23	**6b**	3.28	**6c**	2.26

**Table 3:** Drug likeness parameters predicted by online computational platform (http://www.swissadme.ch/index.php, accessed 29 May 2024).

No.	Mol. Weight [g/mol]	nHA	nHD	Mol. Refr.	TPSA [Å^2^]	Log S (ESOL)
**2a**	230.27	5	1	65.81	65.96	−2.20
**2b**	230.27	5	1	65.81	65.96	−2.20
**2c**	230.27	5	1	65.81	65.96	−2.20
**3a**	280.32	3	1	81.15	65.96	−3.17
**3b**	280.32	3	1	81.15	65.96	−3.17
**3c**	280.32	3	1	81.15	65.96	−3.17
**4a**	325.32	5	1	89.98	111.78	−3.20
**4b**	325.32	5	1	89.98	111.78	−3.20
**4c**	325.32	5	1	89.98	111.78	−3.20
**5a**	312.39	3	1	87.91	91.26	−3.69
**5b**	312.39	3	1	87.91	91.26	−3.69
**5c**	312.39	3	1	87.91	91.26	−3.69
**6a**	300.74	3	1	81.75	65.96	−3.80
**6b**	300.74	3	1	81.75	65.96	−3.80
**6c**	300.74	3	1	81.75	65.96	−3.80

Log S (S expressed in mol/L).

**Table 4:** The HOMO and LUMO orbital energies dipole moment values the energy gap between HOMO and LUMO orbitals calculated for all studied compounds.

No.	Orbital Energy HOMO [eV]	Orbital Energy LUMO [eV]	Dipole Moment [D]	Energy Gap [eV]
**2a**	−4.9637	−1.6533	4.2802	−3.3104
**2b**	−5.2402	−1.5735	3.2400	−3.6667
**2c**	−4.8220	−1.5664	2.9437	−3.2556
**3a**	−4.9470	−1.7802	4.5730	−3.1668
**3b**	−5.2690	−1.7091	4.2067	−3.5599
**3c**	−4.8245	−1.6968	3.5580	−3.1277
**4a**	−5.1137	−3.8957	5.0464	−1.2180
**4b**	−5.3183	−3.9172	4.9282	−1.4011
**4c**	−4.9897	−3.8900	5.5206	−1.0997
**5a**	−4.9745	−1.7421	4.6565	−3.0741
**5b**	−4.8162	−1.5520	3.1396	−3.4225
**5c**	−4.8162	−1.5058	4.4182	−3.4104
**6a**	−5.0682	−2.6174	3.1664	−2.4508
**6b**	−5.3628	−2.5551	2.6640	−2.8077
**6c**	−4.9395	−2.5464	1.9501	−2.3931

**Table 5:** Correlation coefficient values between the lipophilicity parameter values obtained experimentally and using theoretical methods for the tested compounds.

	iLOGP	XLOGP3	WLOGP	MLOGP	SILICOS-IT	Cons. LogP_o/w_	miLogP	ALOGPs	XLOGP2	KOWWIN	ACD/LogP	logPTLC
iLOGP	1.000	0.784	0.474	0.763	0.877	0.888	0.680	0.459	0.702	0.677	0.823	0.514
XLOGP3		1.000	0.886	0.977	0.606	0.974	0.911	0.873	0.975	0.920	0.940	0.693
WLOGP			1.000	0.905	0.180	0.778	0.899	0.996	0.944	0.911	0.817	0.744
MLOGP				1.000	0.561	0.960	0.910	0.902	0.986	0.967	0.956	0.731
SILICOS-IT					1.000	0.757	0.417	0.160	0.484	0.463	0.616	0.209
Cons. LogP_o/w_						1.000	0.864	0.764	0.933	0.897	0.945	0.643
miLogP							1.000	0.877	0.939	0.914	0.900	0.741
ALOGPs								1.000	0.930	0.899	0.822	0.756
XLOGP2									1.000	0.979	0.914	0.718
KOWWIN										1.000	0.879	0.695
ACD/LogP											1.000	0.789
logPTLC												1.000

**Table 6:** Correlation equation and parameters describing them, for relationships between logP_TLC_ and other lipophilicity parameters.

Parameter	Equation	r	s	F	*p*
XLOGP2	LogP_TLC_ = 0.889(±0.239) XLOGP2 + 0.060(±0.582)	0.718	0.522	14	0.003
XLOGP3	LogP_TLC_ = 0.844(±0.244) XLOGP3 + 0.338(±0.546)	0.693	0.541	12	0.004
WLOGP	LogP_TLC_ = 0.951(±0.237) WLOGP − 0.198(±0.603)	0.744	0.501	16	0.002
MLOGP	LogP_TLC_ = 1.052(±0.272) MLOGP + 0.191(±0.528)	0.731	0.512	15	0.002
miLogP	LogP_TLC_ = 0.982(±0.247) miLogP − 0.277(±0.628)	0.741	0.504	16	0.002
ACD/logP	LogP_TLC_ = 0.817(±0.177) ACD/logP + 0.731(±0.332)	0.789	0.461	21	0.001
KOWWIN	LogP_TLC_ = 1.339(±0.384) KOWWIN − 1.339(±0.384)	0.695	0.539	12	0.004
ALOGPs	LogP_TLC_ = 1.202(±0.289) ALOGPs − 0.772(±0.718)	0.756	0.491	17	0.001
Cons.LogP_o/w_	LogP_TLC_ = 0.996(±0.330) Cons.LogP_o/w_ + 0.084(±0.705)	0.642	0.574	9	0.010

## Data Availability

The original contributions presented in the study are included in the article/Supplementary Materials, further inquiries can be directed to the corresponding author/s.

## Supplementary Materials

The following supporting information can be downloaded at: https://www.mdpi.com/article/10.3390/ph17111476/s1, Figure S1: The linear regression between the literature lipophilicity and experimentally obtained R_M0_ for standard substances; Table S1: Values of lipophilicity parameters R_M0_ i logP_TLC_ determined for compounds **2**(**a**–**c**)–**6**(**a**–**c**); Table S2: Numbering of compounds used for research, chemical names and SMILES corresponding to individual structures; Table S3: Theoretical values of the lipophilicity parameter calculated using various algorithms; Table S4: Pharmacokinetic assessment (adsorption, distribution and metabolism parameters) of test compounds **2**(**a**–**c**)–**6**(**a**–**c**) performed using the admetSAR 2.0 web server; Table S5: Selected toxicity parameters of test compounds **2**(**a**–**c**)–**6**(**a**–**c**) performed using the admetSAR 2.0 web server.
