# Supplementary material for: Aniline Derivatives Containing 1-Substituted 1,2,3-Triazole System as Potential Drug Candidates: Pharmacokinetic Profile Prediction, Lipophilicity Analysis Using Experimental and In Silico Studies
Source: Pharmaceuticals (Basel). 2024 Nov 2;17(11):1476. doi: 10.3390/ph17111476 (PMC11597839; doi:10.3390/ph17111476)
Supplement: Supplementary file 1 [file pharmaceuticals-17-01476-s001.zip › pharmaceuticals-3272668-supplementary.pdf]

Supplementary Materials

Aniline Derivatives Containing 1-Substituted 1,2,3-Triazole System as Potential Drug Candidates: Pharmacokinetic Profile Prediction, Lipophilicity Analysis Using Experimental and *in silico* Studies

Elwira Chrobak [1,*], Katarzyna Bober-Majnusz [2] Mirosław Wyszomirski [3] and Andrzej Zięba [1,*]

Figure S1. The linear regression between the literature lipophilicity and experimentally obtained R_{M0} for standard substances.

Table S1. Values of lipophilicity parameters R_{M0} i $logP_{TLC}$ determined for compounds **2(a-c)-6(a-c)**.

Table S2. Numbering of compounds used for research, chemical names and SMILES corresponding to individual structures.

Table S3. Theoretical values of the lipophilicity parameter calculated using various algorithms.

Table S4. Pharmacokinetic assessment (adsorption, distribution and metabolism parameters) of test compounds **2(a-c)-6(a-c)** performed using the admetSAR 2.0 web server.

Table S5. Selected toxicity parameters of test compounds **2(a-c)-6(a-c)** performed using the admetSAR 2.0 web server.

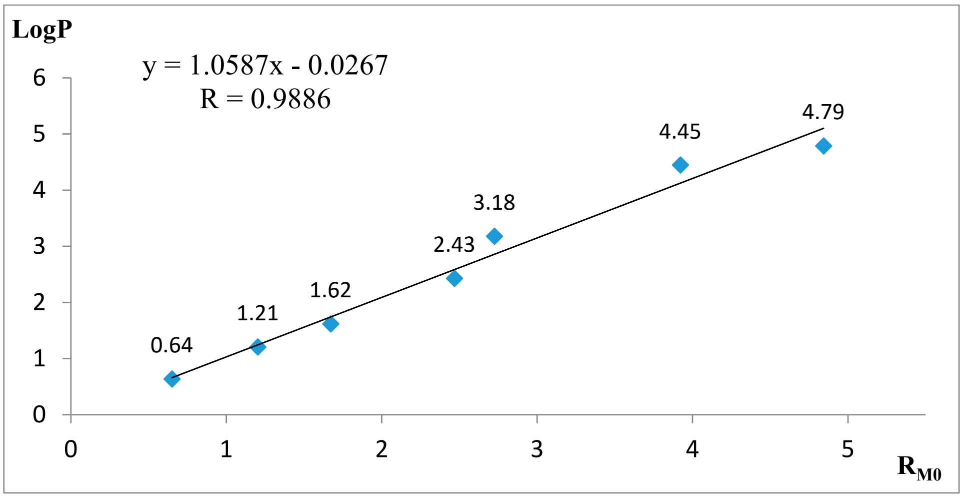

Figure S1. The linear regression between the literature lipophilicity and experimentally obtained R_{M0} for standard substances.

Table I1. Values of lipophilicity parameters R_{M0} i $logP_{TLC}$ determined for compounds **2(a-c)-6(a-c)**.

No	Structure	R_{M0}	*-b*	*r*	$logP_{TLC}$
**2a**	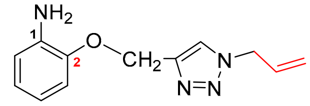	1.194	0.022	0.999	1.24
**2b**	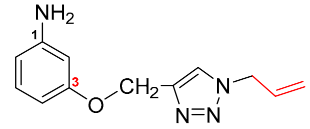	1.301	0.025	0.997	1.35
**2c**	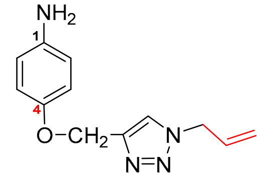	1.109	0.023	0.991	1.15
**3a**	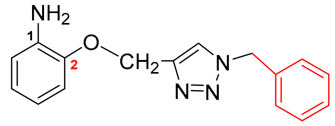	2.240	0.034	0.993	2.35
**3b**	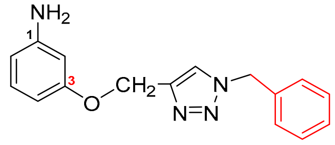	2.197	0.035	0.986	2.30
**3c**	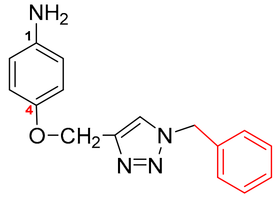	1.246	0.024	0.996	1.29

**4a**	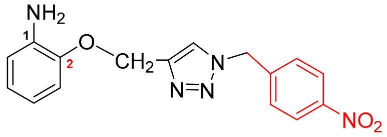	2.542	0.039	0.996	2.66
**4b**	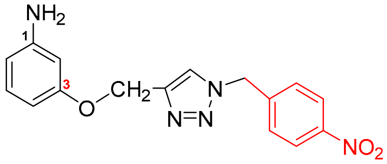	2.618	0.042	0.995	2.74
**4c**	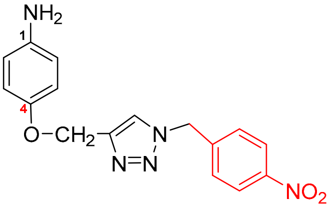	1.600	0.029	0.999	1.67
**5a**	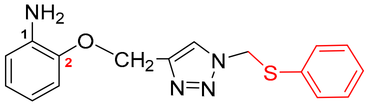	2.564	0.038	0.996	2.69
**5b**	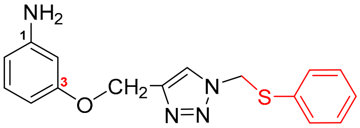	2.502	0.038	0.996	2.62
**5c**	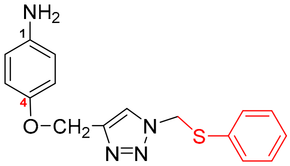	1.606	0.028	0.999	1.67
**6a**	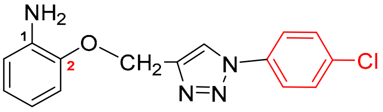	3.072	0.043	0.996	3.23
**6b**	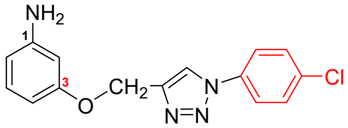	3.121	0.045	0.994	3.28
**6c**	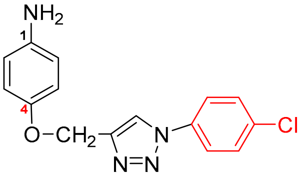	2.162	0.033	0.995	2.26

Table S2. Numbering of compounds used for research, chemical names and SMILES corresponding to individual structures.

Nr zw.	Substituent R	Position in the aniline ring	Chemical name and SMILES
**2a**	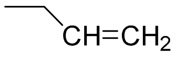	*ortho-*	1-allyl-4-(2-aminophenoxy)methyl-1*H*-1,2,3-triazole<br>NC1=C(OCC2=CN(CC=C)N=N2)C=CC=C1
**2b**		*meta-*	1-allyl-4-(3-aminophenoxy)methyl-1*H*-1,2,3-triazole<br>C=CCN1N=NC(COC2=CC(N)=CC=C2)=C1
**2c**		*para-*	1-allyl-4-(4-aminophenoxy)methyl-1*H*-1,2,3-triazole<br>C=CCN1N=NC(COC2=CC=C(N)C=C2)=C1
**3a**	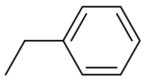	*ortho-*	1-benzyl-4-(2-aminophenoxy)methyl-1*H*-1,2,3-triazole<br>NC1=C(OCC2=CN(CC3=CC=CC=C3)N=N2)C=CC=C1
**3b**		*meta-*	1-benzyl-4-(3-aminophenoxy)methyl-1H-1,2,3-triazole<br>NC1=CC=CC(OCC2=CN(CC3=CC=CC=C3)N=N2)=C1
**3c**		*para-*	1-benzyl-4-(4-aminophenoxy)methyl-1*H*-1,2,3-triazole<br>NC1=CC=C(OCC2=CN(CC3=CC=CC=C3)N=N2)C=C1
**4a**	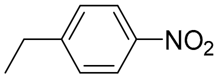	*ortho-*	1-(4-nitrobenzyl)-4-(2-aminophenoxy)methyl-1H-1,2,3-triazole<br>NC1=C(OCC2=CN(CC3=CC=C([N+]([O-])=O)C=C3)N=N2)C=CC=C1
**4b**		*meta-*	1-(4-nitrobenzyl)-4-(3-aminophenoxy)methyl-1*H*-1,2,3-triazole<br>NC1=CC=CC(OCC2=CN(CC3=CC=C([N+]([O-])=O)C=C3)N=N2)=C1
**4c**		*para-*	1-(4-nitrobenzyl)-4-(4-aminophenoxy)methyl-1*H*-1,2,3-triazole<br>NC1=CC=C(OCC2=CN(CC3=CC=C([N+]([O-])=O)C=C3)N=N2)C=C1
**5a**	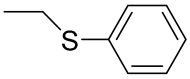	*ortho-*	1-(phenylthio)methyl-4-(2-aminophenoxy)methyl-1*H*-1,2,3-triazole<br>NC1=C(OCC2=CN(CSC3=CC=CC=C3)N=N2)C=CC=C1
**5b**		*meta-*	1-(phenylthio)methyl-4-(3-aminophenoxy)methyl-1*H*-1,2,3-triazole<br>NC1=CC=CC(OCC2=CN(CSC3=CC=CC=C3)N=N2)=C1
**5c**		*para-*	1-(phenylthio)methyl-4-(3-aminophenoxy)methyl-1*H*-1,2,3-triazole<br>NC1=CC=C(OCC2=CN(CSC3=CC=CC=C3)N=N2)C=C1
**6a**	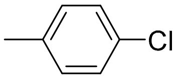	*ortho-*	1-(4-chlorophenyl)-4-(2-aminophenoxy)methyl-1*H*-1,2,3-triazole<br>NC1=C(OCC2=CN(C3=CC=C(Cl)C=C3)N=N2)C=CC=C1
**6b**		*meta-*	1-(4-chlorophenyl)-4-(3-aminophenoxy)methyl-1*H*-1,2,3-triazole<br>ClC1=CC=C(N2N=NC(COC3=CC(N)=CC=C3)=C2)C=C1
**6c**		*para-*	1-(4-chlorophenyl)-4-(4-aminophenoxy)methyl-1*H*-1,2,3-triazole<br>ClC1=CC=C(N2N=NC(COC3=CC=C(N)C=C3)=C2)C=C1

Table S3. Theoretical values of the lipophilicity parameter calculated using various algorithms.

Compound	iLOGP	XLOGP3	WLOGP	MLOGP	SILICOS-IT	Consensus $LogP_{o/w}$	miLogP	ALOGPs	XLOGP2	KOWWIN	ACD/LogP
**2a**	2.07	1.25	1.48	1.04	1.25	1.42	1.82	1.65	1.34	1.68	0.97
**2b**	2.09	1.25	1.48	1.04	1.25	1.42	1.44	1.64	1.34	1.68	0.71
**2c**	2.22	1.25	1.48	1.04	1.25	1.45	1.46	1.66	1.34	1.68	0.62
**3a**	2.53	2.10	2.34	1.93	1.99	2.18	2.77	2.30	2.43	2.54	1.92
**3b**	2.33	2.10	2.34	1.93	1.99	2.14	2.39	2.31	2.43	2.54	1.66
**3c**	2.52	2.10	2.34	1.93	1.99	2.18	2.41	2.30	2.43	2.54	1.57
**4a**	2.03	1.93	2.78	1.75	0.20	1.74	2.73	2.69	2.32	2.36	1.65
**4b**	2.09	1.93	2.78	1.75	0.20	1.75	2.35	2.70	2.32	2.36	1.39
**4c**	1.98	1.93	2.78	1.75	0.20	1.73	2.37	2.71	2.32	2.36	1.30
**5a**	2.74	2.75	2.89	2.20	2.08	2.53	3.22	2.70	2.85	2.59	2.33
**5b**	2.47	2.75	2.89	2.20	2.08	2.48	2.84	2.71	2.85	2.59	2.07
**5c**	2.42	2.75	2.89	2.20	2.08	2.47	2.87	2.72	2.85	2.59	1.98
**6a**	2.76	2.80	2.94	2.47	2.25	2.64	3.13	2.86	2.91	2.69	2.93
**6b**	2.67	2.80	2.94	2.47	2.25	2.63	2.75	2.86	2.91	2.69	2.67
**6c**	2.56	2.79	2.94	2.47	2.25	2.60	2.77	2.87	2.91	2.69	2.58

Table S4. Pharmacokinetic assessment (absorption, distribution and metabolism parameters) of test compounds **2(a-c)-6(a-c)** performed using the admetSAR 2.0 web server.

	Parameter	2a	2b	2c	3a	3b	3c	4a	4b	4c	5a	5b	5c	6a	6b	6c
**A**	HIA	**0.9924**	**0.9953**	**0.9953**	**0.9935**	**0.9961**	**0.9961**	**0.9868**	**0.9912**	**0.9912**	**0.9838**	**0.9897**	**0.9897**	**0.9966**	**1.0000**	**1.0000**
	Caco-2	**0.4909**	0.6566	0.5474	**0.5591**	0.6185	**0.6015**	0.5630	0.6921	0.5541	0.6542	0.7142	0.6828	**0.5465**	**0.5457**	**0.5436**
	HOB	**0.7000**	**0.7143**	**0.7143**	**0.6429**	**0.6429**	**0.6429**	**0.7000**	**0.6857**	**0.7429**	**0.5571**	**0.5571**	**0.5000**	**0.7571**	**0.7714**	**0.7857**
**D**	BBB	**0.7750**	**0.8250**	**0.8250**	**0.7500**	**0.8250**	**0.8250**	**0.7250**	**0.7500**	**0.7500**	**0.8000**	**0.8500**	**0.8500**	**0.8537**	**0.8750**	**0.8750**
	OATP inhibitors:															
	OATP 2B1	1.0000	1.0000	1.0000	1.0000	1.0000	1.0000	1.0000	1.0000	1.0000	1.0000	1.0000	1.0000	1.0000	1.0000	1.0000
	OATP 1B1	**0.9556**	**0.9558**	**0.9634**	**0.9556**	**0.9537**	**0.9607**	**0.9229**	**0.9241**	**0.9364**	**0.9598**	**0.9578**	**0.9633**	**0.9549**	**0.9566**	**0.9649**
	OATP 1B3	**0.9362**	**0.9374**	**0.9374**	**0.9336**	**0.9356**	**0.9356**	**0.9377**	**0.9384**	**0.9384**	**0.9353**	**0.9361**	**0.9361**	**0.9333**	**0.9355**	**0.9355**
	MATE1 inhibitor	0.9400	0.9400	0.9400	0.9400	0.9400	0.9400	0.9400	0.9400	0.9400	0.9400	0.9400	0.9400	0.8600	0.8600	0.8600
	OCT2 inhibitor	0.7000	0.7250	0.7250	0.6500	0.6750	0.6750	0.6750	0.7000	0.7000	0.7000	0.7000	0.7000	0.7250	0.7500	0.7500
	BSEP inhibitor	0.8241	0.7568	0.8541	0.6850	0.4882	0.6876	0.5535	0.6053	0.5856	0.6001	**0.5873**	**0.6360**	0.6328	0.6049	0.6520
	P-glycoprotein:															
	inhibitor	0.9645	0.9552	0.9601	0.8665	0.8147	0.8436	0.6684	0.6850	0.6131	0.7579	0.7259	0.7688	0.8749	0.8609	0.8498
	substrate	1.0000	0.7969	0.8598	0.7021	0.7641	0.8406	0.7364	0.7619	0.8302	0.7369	0.7850	0.8578	0.7548	0.7840	0.8415
**M**	Cytochrome P450:															
	CYP450 3A4 substrate	0.5374	0.5200	0.5668	0.5068	**0.5151**	0.5110	**0.6308**	**0.6372**	**0.6062**	0.5426	0.5137	0.5440	**0.5511**	**0.5638**	**0.5228**
	CYP450 2C9 substrate	1.0000	1.0000	1.0000	1.0000	1.0000	1.0000	0.7861	0.7861	0.7861	0.8086	0.8086	0.8086	1.0000	1.0000	1.0000
	CYP450 2D6 substrate	0.7744	0.7744	0.7744	0.7694	0.7694	0.7694	0.8435	0.8435	0.8435	0.7724	0.7724	0.7724	0.7980	0.7980	0.7980
	CYP450 3A4 inhibition	0.7892	0.8441	0.8441	**0.5108**	0.6615	0.6615	**0.5569**	0.5724	0.5724	**0.6930**	**0.6001**	**0.6001**	**0.8044**	**0.6062**	**0.6062**
	CYP450 2C9 inhibition	**0.5213**	0.5191	0.5191	**0.6510**	**0.6050**	**0.6050**	**0.6453**	**0.5000**	**0.5000**	**0.6816**	**0.6329**	**0.6329**	**0.7639**	**0.7291**	**0.7291**
	CYP450 2C19 inhibition	**0.6332**	**0.5522**	**0.5522**	**0.7869**	**0.7221**	**0.7221**	**0.6800**	**0.5625**	**0.5625**	**0.8107**	**0.7748**	**0.7748**	**0.8277**	**0.7753**	**0.7753**
	CYP450 2D6 inhibition	0.8138	0.8172	0.8172	0.7709	0.7746	0.7746	0.8693	0.8738	0.8738	0.8216	0.8399	0.8399	0.8138	0.8259	0.8259
	CYP450 1A2 inhibition	**0.7227**	**0.6771**	**0.6771**	**0.7556**	**0.7310**	**0.7310**	**0.7299**	**0.6509**	**0.6509**	**0.8231**	**0.7818**	**0.7818**	**0.9103**	**0.9081**	**0.9081**
	CYP inhibitory promiscuity	**0.6920**	**0.5736**	**0.5736**	**0.8883**	**0.8292**	**0.8292**	**0.8793**	**0.8454**	**0.8454**	**0.9424**	**0.9290**	**0.9290**	**0.9496**	**0.9236**	**0.9236**

Abbreviations: HIA - Human Intestinal Absorption; HOB - Human Oral Bioavailability; BBB - Blood-Brain Barrier; OATP - Organic Anion-Transporting Polypeptide; MATE1 - Multidrug And Toxin Extrusion; OCT2 - Organic Cation Transporters; BSEP - Bile Salt Export Pump
bold font - probability of an action occurring

Table S5. Selected toxicity parameters of test compounds **2(a-c)-6(a-c)** performed using the admetSAR 2.0 web server.

	Carcinogenicity (binary)*	Organ toxicity		Terrestrial organism	Aquatic organism
		Hepatotoxicity	Nephrotoxicity	Honey bee toxicity	Fish aquatic toxicity
**2a**	-	+	+	-	-
**2b**	-	+	-	-	-
**2c**	-	+	-	-	-
**3a**	-	+	+	-	+
**3b**	-	+	+	-	+
**3c**	-	+	+	-	+
**4a**	-	+	+	-	+
**4b**	-	+	+	-	+
**4c**	-	+	+	-	+
**5a**	-	+	+	-	+
**5b**	-	+	-	-	+
**5c**	-	+	-	-	+
**6a**	-	+	+	-	+
**6b**	-	+	+	-	+
**6c**	-	+	+	-	+

* binary classification models predict chemical is a carcinogenic or non-carcinogenic
